# Transactions of the New York Pathological Society

**Published:** 1877-08

**Authors:** 


*Transactions of the New York Pathological Society.* Founded in 1844. Volume I. Based on the proceedings for the year 1875, and largely supplemented from the records of 1844 to 1872. John C. Peters, M. D., Editor. Geo. F. Shrady, M. D., E. C. Seguin, M. D., and Thomas E. Satterthwaite, M. D., *Committee on Publication.* New York: Wm. Wood & Co., 1876.

This is the first of a number of volumes which the society has authorized. In it the origin and growth of the society is sketched in brief, from its formation until 1876, following which is a complete list of all who have held office in the society, as well as of the members past and present. The remainder of the work consists of reports of cases and descriptions of specimens. The most rare and interesting are alone presented. the entire work of the society for over thirty years having been subjected to close scrutiny, in order that the most instructing cases might be selected. The editor has put forth a work which will be found to be of more interest than the majority of society reports, and shows from the number of cases cited, and the pains taken to accurately report them that the society is accustomed to have good, solid and conciencious matter presented for its consideration. It is to be hoped that the society will now make up for the lack of any previous publication, by publishing the remainder of its transactions at short intervals, until its mass of material is all before the profession. C.

